# Cold Seep Epifaunal Communities on the Hikurangi Margin, New Zealand: Composition, Succession, and Vulnerability to Human Activities

**DOI:** 10.1371/journal.pone.0076869

**Published:** 2013-10-18

**Authors:** David A. Bowden, Ashley A. Rowden, Andrew R. Thurber, Amy R. Baco, Lisa A. Levin, Craig R. Smith

**Affiliations:** 1 Coasts and Oceans Centre, National Institute of Water and Atmospheric Research, Wellington, New Zealand; 2 College of Earth, Ocean, and Atmospheric Sciences, Oregon State University, Corvallis, Oregon, United States of America; 3 Department of Earth, Ocean and Atmospheric Sciences, Florida State University, Tallahassee, Florida, United States of America; 4 Center for Marine Biodiversity and Conservation, Integrative Oceanography Division, Scripps Institution of Oceanography, La Jolla, California, United States of America; 5 Department of Oceanography, School of Ocean and Earth Science and Technology, University of Hawaii at Manoa, Honolulu, Hawaii, United States of America; Université du Québec à Rimouski, Canada

## Abstract

Cold seep communities with distinctive chemoautotrophic fauna occur where hydrocarbon-rich fluids escape from the seabed. We describe community composition, population densities, spatial extent, and within-region variability of epifaunal communities at methane-rich cold seep sites on the Hikurangi Margin, New Zealand. Using data from towed camera transects, we match observations to information about the probable life-history characteristics of the principal fauna to develop a hypothetical succession sequence for the Hikurangi seep communities, from the onset of fluid flux to senescence. New Zealand seep communities exhibit taxa characteristic of seeps in other regions, including predominance of large siboglinid tubeworms, vesicomyid clams, and bathymodiolin mussels. Some aspects appear to be novel; however, particularly the association of dense populations of ampharetid polychaetes with high-sulphide, high-methane flux, soft-sediment microhabitats. The common occurrence of these ampharetids suggests they play a role in conditioning sulphide-rich sediments at the sediment-water interface, thus facilitating settlement of clam and tubeworm taxa which dominate space during later successional stages. The seep sites are subject to disturbance from bottom trawling at present and potentially from gas hydrate extraction in future. The likely life-history characteristics of the dominant megafauna suggest that while ampharetids, clams, and mussels exploit ephemeral resources through rapid growth and reproduction, lamellibrachid tubeworm populations may persist potentially for centuries. The potential consequences of gas hydrate extraction cannot be fully assessed until extraction methods and target localities are defined but any long-term modification of fluid flow to seep sites would have consequences for all chemoautotrophic fauna.

## Introduction

Cold seeps are sites where fluids enriched with hydrocarbons, primarily methane, emerge from the seabed. They are known from both active and passive continental margins, typically occurring on the continental slope but also much shallower [1]–[3]. The flux of methane-rich fluids at seeps supports distinctive chemoautotrophic food webs in which primary production takes place via microbial oxidation of methane and hydrogen sulphide. Metazoan communities at seeps are characterised by high densities and relatively low diversity of invertebrate taxa which have evolved symbioses with methane- or sulphide-oxidising bacteria, enabling them to exploit the energy potential of the emerging fluids [4].

Although cold seeps are now known to occur on continental margins throughout the global ocean, including the Arctic and Antarctic [5], [6] variations in the composition of seep communities in different parts of the world are not well resolved [7]. Recent research initiatives, particularly those within the Chemosynthetic Ecosystem Science (ChEsS) project of the Census of Marine Life [5] have sought to improve understanding of the biogeography of seep faunas by focusing attention on areas of the oceans that have been relatively under-sampled. Concern has also been raised about the threats that seep sites and their communities face from trawling and future mining for gas hydrates [8], [9], and the need to provide relevant ecological information to inform management strategies for protection of potentially vulnerable seep communities [10].

New Zealand was one of four regions targeted by the ChEsS programme. The existence of cold seeps around New Zealand was initially deduced from fisheries by-catch and research trawl and dredge samples [11]. The first dedicated biological sampling of seep sites on the Hikurangi Margin off the east coast of the North Island took place in 2006 with the “New Zeeps” voyage (TAN0616) [8] and was continued during geophysically-focused voyages in 2007 (SO191) and 2011 (SO214). To date, at least thirty-two sites of active seepage have been identified on the Hikurangi Margin at depths of 600 to 2000 m [12]. All known seep sites on the Hikurangi Margin are methane-derived authigenic carbonate mounds (sensu [13]). Mud volcanoes are apparently absent from the margin and no evidence of chemoautotrophic fauna has been recovered from the few areas of pockmarks that have been detected to date in the region [12]. Megafaunal communities associated with the seep sites are dominated by vesicomyid clams, large siboglinid tubeworms, and bathymodiolin mussels [8], [14], [15]. White bacterial mats and dark sulphide-rich patches which host dense populations of ampharetid polychaetes are characteristic on soft sediments around carbonate concretions [16]–[18], and frenulate siboglinid worms have been found in high densities on the peripheries of some seep sites [17].

Baco et al. [8] presented initial descriptions of community composition and habitats at eight Hikurangi Margin seep sites based on data from towed camera, epibenthic sled, grab, and multicorer samples collected during TAN0616. These authors speculated that the seep communities on the Hikurangi margin could represent a distinct biogeographical province but this contention remains untested until the taxonomic and genetic identities of the species sampled have been established. Baco et al. [8] also observed that bottom trawls had impacted several seep sites and highlighted the need for further research on seep ecology to inform environmental management of this and potential future threats to seep communities. The Hikurangi Margin has strong potential for large-scale exploitation of gas hydrates as an energy resource [19]. Although seabed exploration of the Hikurangi Margin gas hydrate province has been focused around sites of active seepage, as identified by the presence of acoustic water column flares and chemoautotrophic fauna [12], geophysical surveys using seismic methods have covered much wider areas of the margin [20]. Published analyses from these surveys provide broader spatial and temporal perspectives on the geophysical context in which the highly localised biological communities exist, providing insight into the processes and pathways controlling the availability of methane-rich fluids at the seabed.

In this study, we review the megafaunal ecology of the known seep sites and, by placing this in the contexts of the likely timescales of ecological processes and present and possible future human impacts, we evaluate the potential vulnerability of the fauna to disturbance. Analysis of all fifty-six camera transects from the three research voyages to the Hikurangi Margin cold seeps enables us to describe and compare seep faunal communities across a total of eighteen sites, each of which has been traversed by at least one camera transect. We do not consider here other reported sites from which seep-associated fauna have been recovered but for which seabed imagery is not available (see [11], [12]). The primary aims of the study are to: (1) describe the principal seep-associated communities and habitats present at seep sites on the Hikurangi margin; (2) assess variability among seep sites in terms of their communities and physical habitats; (3) use observations from the New Zealand seeps and life-history information from the literature to develop a hypothetical succession model for the Hikurangi Margin seeps, and (4) assess the vulnerability of seep communities to current and potential future human activities on this margin.

## Methods

### Study area and sites

**Figure 1:**
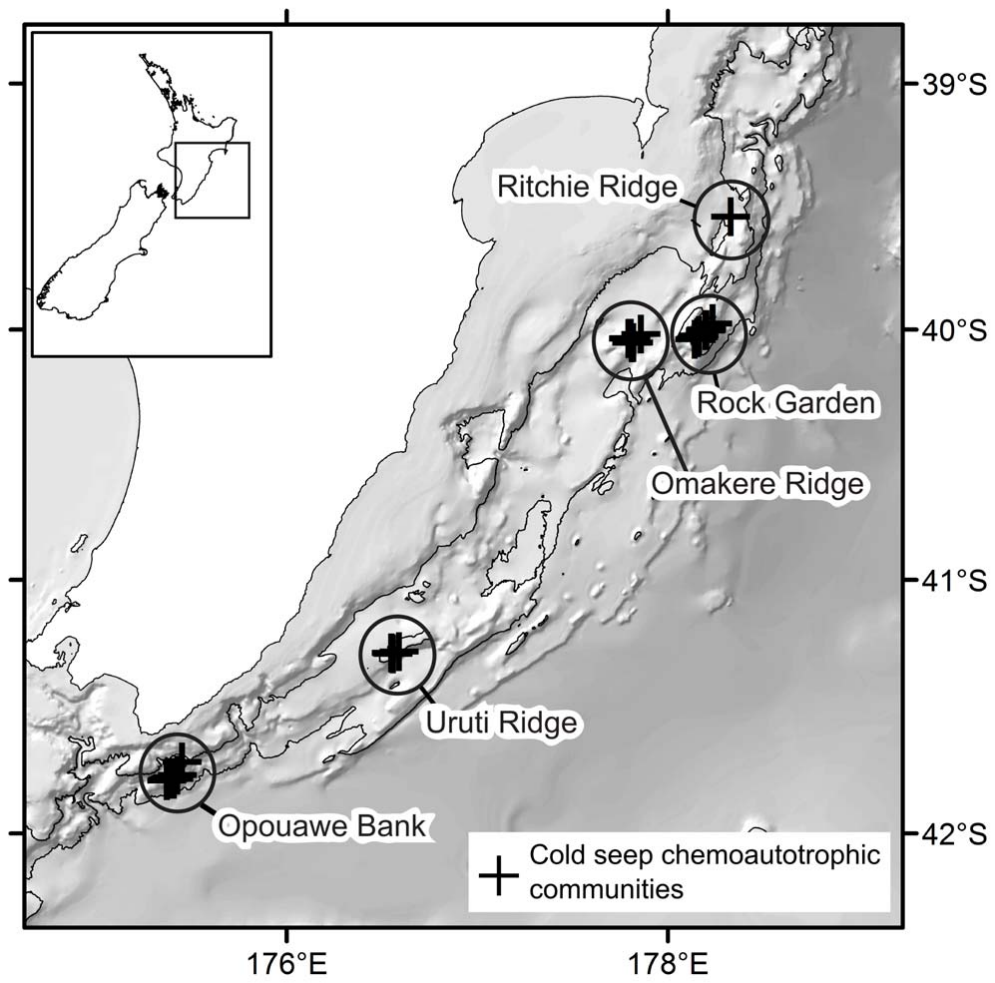
Cold seep sites on the Hikurangi Margin of New Zealand. Labelled circles indicate regions referred to in the text, crosses show individual seep sites at which live chemoautotrophic communities have been sampled (see Table 1 and Greinert et al. [12] for site details).

The Hikurangi Margin is an active subduction zone off the east coast of North Island, New Zealand, representing the southern extension of the Tonga-Kermadec subduction system [20]. Cold seep exploration to date has been concentrated on the seabed from Ritchie Ridge in the north (39°30′S) to Opouawe Bank in the south (41°47′S), and active seeps have been discovered at water depths from ca. 600 to 1200 m. Five principal regions of seep activity have been studied: Ritchie Ridge, Rock Garden, Omakere Ridge, Uruti Ridge, and Opouawe Bank (Figure 1) across which a total of thirty-two active seeps have been confirmed [12]. Evidence of chemoautotrophic faunal communities has been found at eighteen of these sites (Table 1 – note, of the individual sites listed by Greinert et al. [12], we treat the following groups as single named sites: Kea and Kaka; Weka A, B, and C; Faure Sites A and B).

**Table 1:** Cold seep sites on the Hikurangi Margin sampled by towed camera transects.

							Camera station numbers			Analysed	
Region	Site name	Site code	Latitude (S)	Longitude (E)	Depth (m)	Area (ha)	TAN0616	SO191	SO214	Stills	Video
Builder's Pencil	Builder's Pencil	BPL	39°32.636	178°19.944	797	7.0	19, 20, 26, 27, 28, 29			142	00:55:31
Rock Garden	LM-3	LM3	39°58.607	178°14.221	863	0.1		42 [2], 176 [19]		26	01:14:00
	Weka (A to C)	WEK	40°00.286	178°11.905	655	12.0		41 [1a]			01:51:00
	Faure Site (A)	FAU	40°01.960	178°09.401	654	na		41 [1]			00:55:00
	Rock Garden knoll	RGK	40°02.379	178°08.599	767	na	5, 14, 15				01:35:02
Uruti Ridge	Hihi	HIH	41°17.687	176°33.548	744	1.5	66			85	00:37:16
	Kereru	KER	41°17.161	176°35.469	727	1.5	64, 69				00:20:08
	LM-10	LM10	39°24.993	178°24.268	729	1.5	65				00:42:31
Omakere Ridge	Bear's Paw	BPW	40°03.187	177°49.252	1100	2.2		52-3 [5], 81 [7]		25	00:38:52
	Kea & Kaka	KAK	40°02.240	177°47.714	1168	8.1		52-1 [3], 80 [6], 167 [17]			04:59:33
	LM-9	LM9	40°00.603	177°52.358	1150	1.5	44, 55, 56				01:27:05
	Moa reef	MOA	40°03.235	177°48.802	1118	11.0		52-3[5], 81[7], 166[16]			04:21:47
	SW Moa	SWM	40°03.336	177°48.247	1120	12.2		81 [7]	94 [10]	57	00:54:00
Opouawe Bank	North Tower	NTR	41°46.911	175°24.083	1052	6.1	75, 76, 85, 114, 115	106 [9]	41 [1]	163	04:59:43
	South Tower	STR	41°47.300	175°24.521	1056	4.5	75, 77, 87, 117, 119, 120, 124				01:26:46
	Piwakawaka	PIW	41°47.664	175°22.348	1095	2.5		292 [22]	70 [5], 71 [6]	57	00:57:20
	Pukeko	PUK	41°47.153	175°23.465	1060	4.3		105 [8], 155 [15], 272 [21]			01:04:04
	Takahe	TAK	41°46.368	175°25.651	1058	6.5	128	107 [10]	64 [2], 65 [3]	23	00:51:04
	Tui	TUI	41°43.288	175°27.091	815	5.2	129	108 [11], 129 [12], 154 [14]		21	03:24:56

Sampling took place during voyages of *RV Tangaroa* (TAN0616, November 2006) and *RV Sonne* (SO191, February 2007; SO214, April 2011). Table shows; region; seep site names and abbreviations; latitude and longitude (of flare position or seep centre); seabed depth; approximate seabed area; station numbers of all camera transects at each site (numbers in square brackets are sequential deployment numbers for *RV Sonne*'s OFOS camera system), and the numbers of still images and hours of video (hh:mm:ss) analysed from each site.

Multibeam sonar bathymetry maps have been generated for all regions, and for Omakere Ridge and Opouawe Bank extensive high-resolution side-scan sonar (SSS) images of the seabed have also been developed [14], [15]. Areas of high backscatter in these images show the locations of exposed and shallow sub-surface carbonate concretions associated with cold seeps and are an accurate indication of seep extent. Some estimation of the vertical relief of the carbonates can also be made from the intensity of shadow areas in the SSS image [14]. Here, we used SSS maps in a geographic information system to measure the areal extent of seep sites. Where SSS coverage was not available, area was estimated from the length of camera transect in which seep fauna or habitats were recorded and the local topography of the site shown in multibeam sonar maps.

### Sampling

The most intensive sampling of the Hikurangi Margin seep fauna, in terms of both the number of deployments and the number of seep sites sampled, has been by towed camera systems. Cameras were deployed at potential seep sites during each of the three research voyages (TAN0616, SO191, and SO214), yielding a total of fifty-seven transects covering approximately 61,000 linear metres of seabed (Table 1). The intensity of sampling within each region was influenced primarily by the number of active seeps detected. Thus, overall effort was greatest at Opouawe Bank and Omakere Ridge because these regions contained the highest densities of active seeps as indicated by broad-scale acoustic detection of water-column flares [12]. All sampling was conducted under the authority of New Zealand Ministry of Fisheries Special Permits 318 (TAN0616 and SO191) and 421 (SO214) issued to the National Institute of Water and Atmospheric Research (NIWA) for the purposes of investigative research and education.

Two camera systems were used; NIWA's deep towed imaging system (DTIS [21]) on TAN0616, and *RV Sonne*'s Ocean Floor Observation System (OFOS) on SO191 and SO214. Both systems recorded continuous digital colour video with a separate digital still image camera capturing higher-resolution images (“stills”) automatically at either 20 s (TAN0616) or 15 s (SO191 and SO214) intervals. The combination of continuous video and intermittent stills afforded two perspectives on each transect. First, video provided a full record of habitat transitions and megafaunal population densities, including infrequently-occurring fauna and microhabitats (i.e. characteristic combinations of fauna and substrata) likely to be missed in intermittent stills. Second, still images enabled accurate density counts for high-density fauna and quantitative characterisation of habitat structure at smaller spatial scales (<1 m^2^). All cameras were oriented directly downwards, to facilitate quantitative analyses, and pairs of red lasers at 0.2 m spacing, parallel to the optical axis of each camera, were projected onto the seabed for image scaling. All transects were tracked using ultra-short baseline acoustic systems (Simrad HPR on *RV Tangaroa* and Ixsea Posidonia on *RV Sonne*), yielding seabed positions with an accuracy of ca. ±20 m at ca. 2–5 s intervals depending on depth. On TAN0616, digital video was recorded in 1080 50i high definition (HD) format with 8 megapixel (mp) digital stills. For SO191 video was recorded in 720×576 standard definition (SD) format with 4 mp stills. For SO214, both HD and SD video cameras were used simultaneously and 10 mp stills taken but HD and useable stills were only recorded successfully on half of the transects. All transects were run at target camera altitude of 2.5 m and speed of 0.25 to 0.5 ms^−1^. Sections of transects which were outside the target altitude and speed ranges, or where quality was poor for technical reasons, were not used in analyses. These criteria resulted in video frame widths ranging from ca. 1 to 3 m, and still image areas from 1.8 m^2^ to 7.0 m^2^. In total, 59.5 h of video and 10,560 still images were collected across the three voyages.

### Analyses

#### Camera transect analysis

Transects were initially cropped to the section bounded by the first and the last occurrence of seep-associated fauna or substrata (typically clam shells, sulphidic patches, or bacterial mats). Thirty-six video transects were analysed using methods described in Jones et al. [14] and Klaucke et al. [15]. Briefly, cropped transects were reviewed using Ocean Floor Observation Protocol software (OFOP, http://ofop.texel.com) to record the occurrence of all seabed megafauna (>ca. 50 mm) and substratum types. Substrata were recorded as coarse level categories (e.g. “muddy sediment”, “carbonate rock”, see [14]) with modifiers indicating overlying biogenic substrata (e.g. “*Calyptogena* sp. shell”). As described in Jones et al [14], substratum categories were treated as continuous, each recorded observation propagating onward through the video transect record at 1 s intervals until the next substratum observation was made. Fauna were recorded as counts of individuals but densities of *Lamellibrachia* sp. exceeded the analyst's ability to discriminate between individuals in places. At these points the OFOP output file becomes ‘saturated’, representing high population density rather than absolute numbers. For transects where this occurred, the rank order of densities between transects was compared with the more precise counts from the still images and video counts were adjusted where necessary to match the stills rank order. The distributions of seep-associated megafauna and habitats were then plotted against SSS maps, where available, to visualise spatial relationships.

Still images were analysed from eleven transects chosen to be representative of each of the major seep regions. For smaller seep sites (<ca. 200 m across) all useable images along the transect within the site were analysed. For larger sites, because individual seep habitats were highly localised at metre scales, subsets of still images were analysed with the aim of representing all microhabitats present and capturing the full range of population densities, including the highest. Using ImageJ (http://rsbweb.nih.gov/ij/) analysis software, images were first scaled by reference to the laser points projected on the seabed. Principal substratum types were then measured as areas and converted to percentages of the whole image. Fauna were identified to the finest practicable taxonomic resolution and counted. Counts were then standardised to numbers of individuals m^−2^ based on imaged seabed area.

#### Comparisons between seep sites

For sites where still images were analysed in detail, population densities of the principal seep-associated taxa (as individuals m^−2^) were plotted together with substratum type (as % of total seabed area of the image) against distance across the seep site. These profiles illustrate differences in the spatial scales of individual seep sites, variations in the principal fauna present and their population densities, and the spatial relationships between habitats and fauna.

Data from video analyses were used in multivariate analyses to explore variability between seep sites and regions. Counts of seep-associated fauna were standardised to unit area by dividing by the seabed area of the transect (cropped transect distance multiplied by image frame width, which was approximated as 2 m for all transects). Count data were log (*x*+1) transformed to reduce the influence of variations in the high population density regions of the video transects, where absolute counts were less precise (see above). Because substrata counts were effectively continuous (i.e. with a categorical value recorded at every 1 s increment along the transect), they were expressed as proportions of the total transect by dividing the sum of counts recorded for each substratum type by the total number of seconds in the analysed portion of the transect. Thus, faunal data were rendered as counts of individuals m^−2^ and substrata as percentages of the total transect.

Data for seven seep-associated fauna characteristics from the video analyses: *Lamellibrachia* sp. tubeworms; *Calyptogena* sp. live clams; Bathymodiolin mussels; *Calyptogena* sp. shell valves; *Stelletta* sp. sponges; dark sediment patches (a proxy for ampharetid polychaete beds), and bacterial mats, together with observations of thicket-forming scleractinian corals, were used to generate a matrix of Hellinger distances [22] between each pair of transects. Non-metric multidimensional scaling (NMDS) was then used to produce a two-dimensional ordination of relationships between the thirty-six analysed video transects. Hellinger distance was chosen because the data included both abundance and proportion values, but trials using Bray-Curtis similarity showed very similar results. The relative influence of each of the measured faunal variables in the final ordination was visualised by superimposing vectors proportional to their Pearson correlations with the 2-dimensional ordination. The same procedure was used to illustrate correlations with three physical environmental factors: depth; percentage of carbonate rock substrata recorded in video, and the number of trawls per site for the period 1998 to 2005 (see below for details). Formal statistical tests for differences between survey regions were not appropriate because of the limited number of variables and differences in numbers of sites between regions (e.g. only one site at Richie Ridge). Multivariate analyses were run in PRIMER [23].

### Assessing impacts of human activities

#### Trawling

The Hikurangi Margin seep sites have been subject to disturbance from bottom trawling, mainly for orange roughy (*Hoplostethus atlanticus*) and oreo (*Pseudocyttus maculates*), since at least 1989 [8]. Using trawl data compiled for the period 1989 to 2005 [24]. We extended the analysis of trawl frequency at the seep sites conducted by Baco et al [8] to include all sites in the study area from which evidence of chemoautotrophic fauna has been collected. Trawling intensity was calculated as the number of trawl tracks intersecting a 250 m radius circle centred on each of the seep locations detailed by Greinert et al. [12]. In most instances, these locations are the coordinates of the seabed origin of acoustic flares [12] but some are identified as the centre of high backscatter areas in SSS images (e.g. Bear's Paw, at which no water column flares were detected during initial surveys). Analyses were run using XTools Pro in ArcGIS v.10.

#### Gas hydrate extraction

Techniques for seabed gas hydrate extraction have yet to be fully developed, and the optimal locations for exploitation on the Hikurangi Margin remain a subject of debate (e.g. [25]), with current opinion erring towards sandy strata in the back limb areas of thrust ridges (see Barnes et al. [20] for geophysical background). Our assessment here, therefore, was restricted to (1) consideration of relationships between seep sites and patterns of fluid flow through the geological structures underlying them, as understood from recent published interpretations of seismic survey data [20], [25]–[28] and (2) the proximity of known seep communities to potential drill sites currently under discussion.

## Results

### Fauna & habitats

With the exception of Takahe, all seep sites were associated with authigenic carbonate rock structures visible at the sediment surface. Photographs and physical samples indicated two broad categories of carbonate formation: light-brown blocks embedded in soft sediments, usually associated with high densities of chemoautotrophic fauna, and referred to as “chemoherm” [14], [29] or “fresh” [30]; and darker, more extensive, “weathered-looking” [30] rock associated with larger geomorphologic structures, sessile heterotrophic taxa and few, if any, chemoautotrophic fauna.

**Figure 2:**
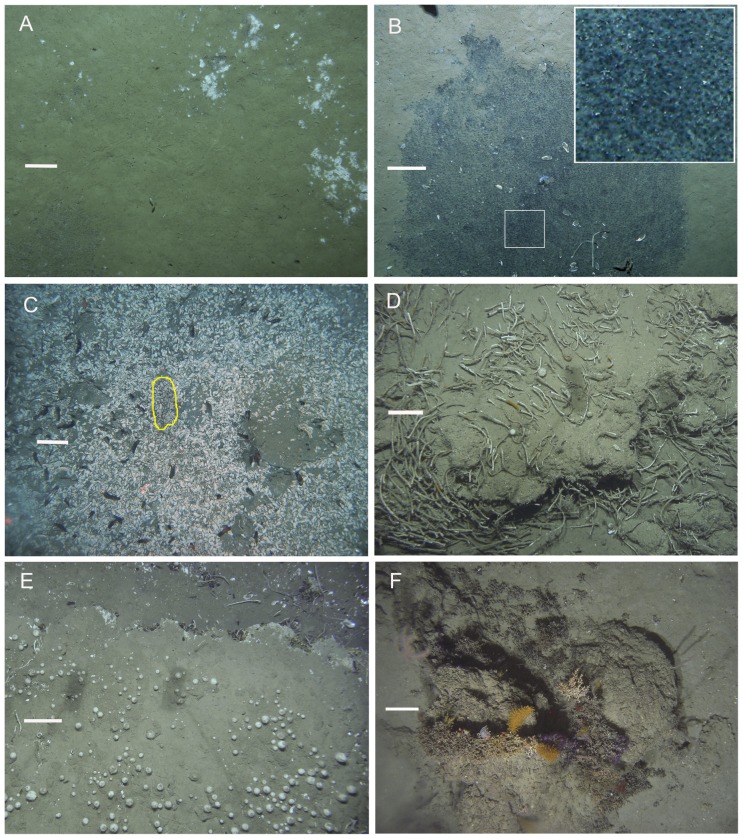
Hikurangi Margin cold seep habitats and fauna (1). (A) bacterial mats on soft sediments at North Tower; (B) sulphide-rich sediment patch with high density ampharetid polychaete population and (inset) characteristic ‘raindrop’ patterning at North Tower; (C) extensive *Calyptogena* sp. shell valves surrounding a population of live clams (yellow outline) with live and dead *Bathymodiolus* sp. mussels at Tui; (D) high density *Lamellibrachia* sp. tubeworm population at Southwest Moa showing tubes emerging from upper surface of carbonate concretions; (E) high densities of *Stelletta* n. sp. sponges and *Bathymodiolus* sp. mussels (along ledge at top) at Southwest Moa, and (F) scleractinian and gorgonian corals on weathered carbonates at Moa. Scale bars show 0.2 m.

**Figure 3:**
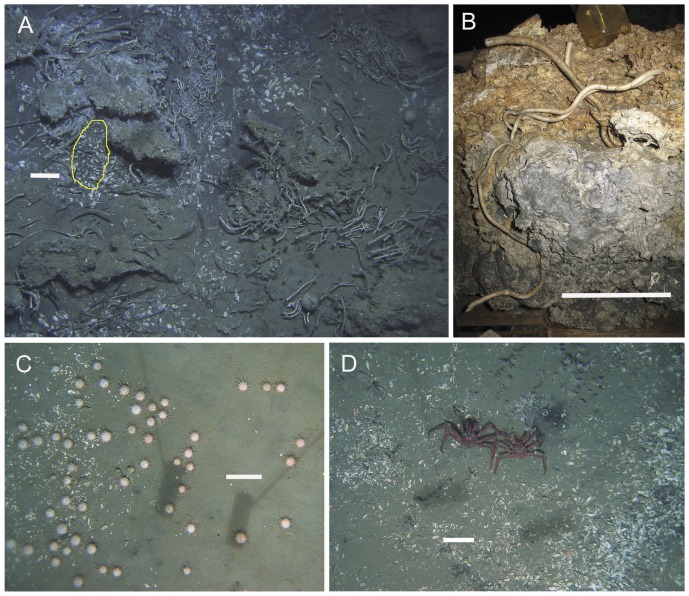
Hikurangi Margin cold seep habitats and fauna (2). (A) ‘Chemoherm’ habitat at Southwest Moa showing *Lamellibrachia* sp. tubeworms in association with authigenic carbonates, live *Calyptogena* sp. clams (yellow outline) and disarticulated shell valves, two spherical *Stelletta* n. sp. sponges, grey bacterial mats on carbonates (at upper left), and sulphidic sediments; (B) Underside of a carbonate block recovered by grab from chemoherm at North Tower, showing *Lamellibrachia* sp. tubes rooted in soft, grey, sulphide-rich, anoxic material at the base of the block; (C) *Gracilechinus multidentatus* sea urchins with *Calyptogena* sp. shell debris on soft sediments at the periphery of Tui seep; (D) *Lithodes aotearoa* crabs amongst *Calyptogena* sp. shell debris at Tui seep. Scale bars show 0.2 m.

At the optical resolution of towed camera transects, seep sites were characterised by communities dominated by five conspicuous metazoan epifaunal taxa: vesicomyid clams in the genus *Calyptogena*; siboglinid tubeworms in the genus *Lamellibrachia*; mussels in the sub-family *Bathymodiolinae*, and sponges in the genera *Pseudosuberites* and *Stelletta* (Figure 2). In addition to these, white bacterial mats and dark sediment patches colonised by ampharetid polychaete worms were conspicuous and locally common (Figure 2 A and B). Individual ampharetids were not visible in images but their populations are known to be strongly associated with these dark, sulphide-rich sediments, in which they form a characteristic ‘rain drop’ patterning of small pits visible at larger scales (Figure 2 B) [8], [16], [17], [31]. These taxa were present in varying combinations across the study area, forming a limited spectrum of characteristic combinations of physical habitat and faunal community within and around active seep sites. Although other chemoautotrophic taxa, including frenulate siboglinid worms and *Acharax* sp. bivalves, were certainly present at many, if not all, sites [8], [17] these were not reliably recorded in camera transects because of their small size and infaunal habit. Four mobile heterotrophic invertebrate taxa characteristic of surrounding slope habitats were also commonly observed within and around seep sites: lithodid crabs were seen at the edges of sulphidic patches containing *Calyptogena* clams (Figure 3 D); pagurid crabs were common on carbonate and sediment substrata at Hihi, Builder's Pencil and North Tower; predatory gastropod molluscs (mostly *Fusitriton* sp.) were common, particularly at Piwakawaka, North Tower, Bear's Paw, and Tui, and the regular echinoid *Gracilechinus multidentatus* occurred in high densities around the periphery of Tui (Figure 3 C).

Dark sediment patches with the distinctive pitting caused by ampharetid polychaetes were observed at all seep sites where soft sediments were present. The pitting was observed in all areas of sulphide-rich sediments, both inside and outside the authigenic carbonate chemoherm of the seeps, and whether colonised by clams or not. Isolated ampharetid patches on open sediments occurred most frequently to the southeast of North Tower, at Takahe, and between Bear's Paw and Moa, with recorded patch size up to ca. 2 m^2^. Bacterial mats were also most common in these areas and were often, but not always, associated with the dark sediment patches. Mats were generally smaller than the dark sediment patches, typically ca. 0.2 m^2^ to the southeast of North Tower but up to ca. 1 m^2^ at Takahe.

Past or present occurrence of *Calyptogena* sp. clams at all sites was indicated by disarticulated shell valves on the seabed (Figures 2 & 3), ranging from small, highly localised patches (<1 m^2^) on muddy sediments at Takahe to ∼70,000 m^2^ of mostly carbonate rock covered by shell valves and fragments at Builder's Pencil. Live clams were identifiable as they were intact (i.e. with valves joined and tightly closed) and in characteristic living position, with only their posterior end protruding from the sediment (see Figure 2 C in [8]). Clam shells were typically 40 to 50 mm in length but individuals reached 80 mm. Shell morphologies indicate that *Calyptogena tuerkayi* is the predominant vesicomyid clam species at all sites (Elena Krylova personal communication) but two valves recovered from LM-9 (station TAN0616-045) suggest that a species of *Laubericoncha* may also be present. Live clams occurred only in highly localised patches (<1 m^2^) of closely packed individuals and were always associated with sulphide-rich sediments with ampharetid pits and surrounded by larger areas of disarticulated valves (Figure 2C). The maximum recorded density of live *Calyptogena* sp. in a single image was 24 individuals m^−2^ at Piwakawaka on Opouawe Bank but because patches of live clams were always somewhat smaller than the total image area, actual densities within patches will be considerably greater than this.

Lamellibrachid tubeworms occurred only in areas where carbonate rocks were present, and appeared to be the same species at all sites. Initial molecular studies suggest the species is *Lamellibrachia columna* (AB, unpublished data), first described from the Lau Basin at the northern end of the Tonga-Kermadec subduction system [32]. Tubes were smooth with indistinct annular rings in places, typically 10 to 15 mm diameter at the anterior opening, and with up to ∼1 m but more usually 0.2–0.5 m visible above the substratum. The anterior portion of the tube was generally straight or slightly curved, lying parallel to the substratum or rising at a shallow angle, and becoming more convoluted at the seabed and within carbonates (Figure 2 D). An incomplete tube extracted from a carbonate block sampled by grab at North Tower (SO191 station 138 TV-G5) had a 16 mm anterior tube diameter and was 1.32 m long, two thirds of this length being within the carbonate block (Figure 3 B). Anterior sections of tubes were often a pale off-white colour in images, presumably representing recent growth, but older parts were usually overgrown with epifauna, including hydroids and sponges (see Figure 2 A in [8]). Tubeworms occurred in loose aggregations, often with their tubes aligned with each other, but not forming the dense ‘thickets’ documented for *L. luymesi* in the Gulf of Mexico [33]. At most sites, tubes were seen only in crevices or under ledges, with none projecting above the upper surface of carbonate blocks or pavements. At Omakere Ridge sites, however, particularly Southwest Moa, tubes were seen emerging directly from the upper surfaces of carbonates (Figure 2 D). Occurrence was patchy within seep sites, with maximum recorded density in a single image of 51 individuals m^−2^ at Southwest Moa.

Live bathymodiolin mussels were recorded at eight of the eighteen sites from which video transects were analysed, and were observed in all regions except Uruti Ridge. Mussels ranged from ca. 50 to 100 mm in length and most appeared to be *Bathymodiolus tangaroa*, based on shell morphology [8 and references therein]. However, at least three species of bathymodiolin have been sampled from the Hikurangi seeps [8], so diversity in this group is likely to be higher than represented here. All live mussels observed were attached to carbonate rocks; highest densities occurring at Southwest Moa (31 individuals m^−2^, Figure 2 E) and in a single small patch within extensive carbonate platforms at LM-3 (77 individuals m^−2^). At Builder's Pencil and Tui, mussels occurred in isolated clusters of a few individuals, often amongst extensive areas of disarticulated *Calyptogena* sp. shells.

The sponges *Pseudosuberites* sp. and *Stelletta* n. sp. (Michelle Kelly, NIWA, unpublished data) were found only on ‘fresh’ carbonate chemoherm within the central areas of seep sites and both taxa reached highest densities in areas where tubeworms and mussels were also abundant. Whereas the encrusting species *Pseudosuberites* sp. was observed at most sites where fresh carbonates and chemoautotrophic megafauna were present, the spherical sponge *Stelletta* n. sp. was recorded only at Omakere Ridge seep sites, notably Southwest Moa, reaching maximum densities of 50 individuals m^−2^.

### Site descriptions

#### Opouawe Bank

**Figure 4:**
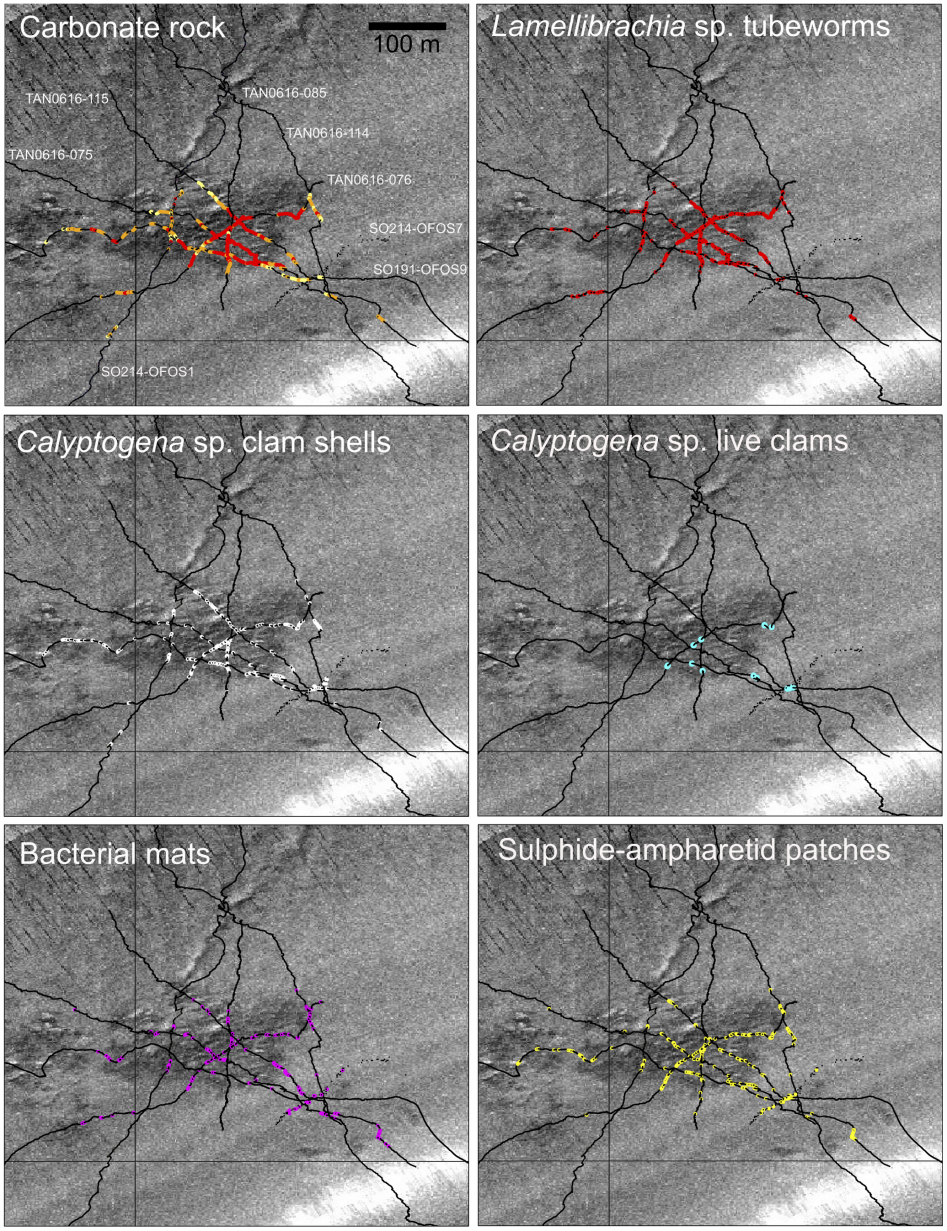
North Tower seep, Opouawe Bank. Observations from towed video camera transects showing distributions of: authigenic carbonate rocks (red, “chemoherm”; orange, “large blocks (>20 cm)”; yellow, “small blocks (<20 cm)”); *Lamellibrachia* sp. siboglinid tubeworms; disarticulated shell valves of *Calyptogena* sp. vesicomyid clams; live *Calyptogena* sp. clams; bacterial mats, and dark, sulphide-rich sediment patches colonised by ampharetid polychaetes. Transects are plotted against a side-scan sonar image of the seabed generated during SO191-2 (darker pixels indicate stronger acoustic backscatter). Labels in the top left panel show voyage and deployment number for each transect: TAN0616, *RV Tangaroa* 2006; SO191, *RV Sonne* 2007; SO214, *RV Sonne* 2011.

**Figure 5:**
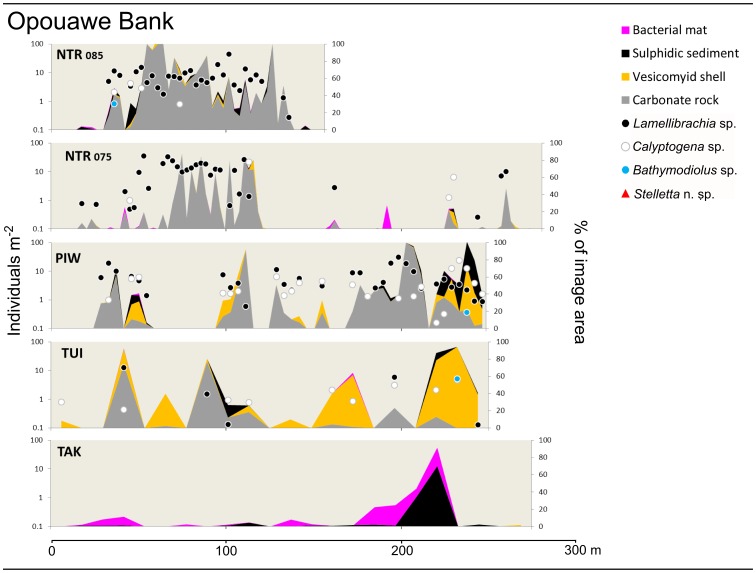
Representative profiles across selected seep sites at Opouawe Bank. Site name abbreviations: NTR, North Tower; PIW, Piwakawaka; TUI, Tui; TAK, Takahe (numbers on North Tower profiles show station numbers; see Figure 4. Station numbers for other sites: PIW, SO214-070; TUI, SO191-129; TAK, SO214-065). Data are from analyses of still images taken during towed camera transects across each site. Point markers show population densities of seep-associated fauna (individuals m^−2^, log scale, left y-axis), area fills show substratum type as proportion of the full image area (% of image area, right y-axis). Profiles are scaled to horizontal distance travelled (x axis).

*North Tower* is the most intensively sampled of the Hikurangi sites [8], [12], [15], [17] and illustrates most of the seep-associated habitats and fauna characteristic of the region (Figure 4). The first signs of seep-associated habitats at the periphery of the seep were scattered *Calyptogena* sp. shells and, on the southeast approaches especially, small patches of white bacterial mat (cm scale) and cm to m scale ampharetid patches. Authigenic carbonates occurred more frequently approaching the outskirts of the high backscatter SSS region and were often associated with low densities of *Lamellibrachia* sp. tubeworms, sometimes protruding directly from the sediment but always in close proximity to carbonate rocks. At the boundary of the high-backscatter region, there was an abrupt transition from predominantly muddy sediments to authigenic carbonate blocks interspersed with dark sediments populated by ampharetids. Tubeworms were strongly associated with carbonates within the seep site (Figure 5). Vesicomyid clam shells covered the seabed in localised patches (m^2^ scale) but live *Calyptogena* sp. were restricted to infrequent, small (<1 m^2^ scale), populations occurring in patches of dark sediments.

*South Tower*. No additional biological sampling has been undertaken at South Tower since TAN0616, findings from which are described by Baco et al. [8] and Klaucke et al [15]. Review of video and stills from the site in the present study confirmed habitats and fauna similar to those recorded at North Tower.

*Piwakawaka* showed in SSS images as a region of stronger backscatter covering a seabed area of ca. 2.45 ha. The site is similar to North Tower in terms of both substrata and fauna (Figure 5), but with low densities of bathymodiolin mussels in places.

*Tui* forms the summit of a rise in the northern part of Opouawe Bank. The site was similar to North Tower in that the central area consisted of exposed authigenic carbonate blocks interspersed by muddy sediments, with tubeworms, clams, and ampharetid patches patchily distributed among them. It differed, however, in having a greater proportion of the substratum covered by *Calyptogena* sp. shells, more live *Calyptogena* sp. patches, and localised populations of bathymodiolin mussels. On muddy sediments surrounding the site, areas of disarticulated clam shells were common and often associated with populations of *G. multidentatus* (Figure 4 C).

*Takahe* showed in SSS images as a roughly circular area of elevated backscatter ca. 250 m in diameter but no carbonates were visible at the seabed. Patches of dark sediment with high densities of ampharetid polychaete pits and white bacterial mats were common (Figure 5), particularly on the north-eastern circumference of the site. Infrequent patches of *Calyptogena* sp. shell were recorded but live clams were seen in only one image. No other chemoautotrophic megafauna were observed. On the last occasion this site was sampled (SO214, April 2011), the dark, sulphide-rich sediments were less distinct in camera transects than in earlier years (2006, 2007). Subsequent core samples showed that the sulphide-rich patches were overlain by deposits of fresh planktonic detrital matter, which obscured the dark sediments below.

#### Uruti Ridge

**Figure 6:**
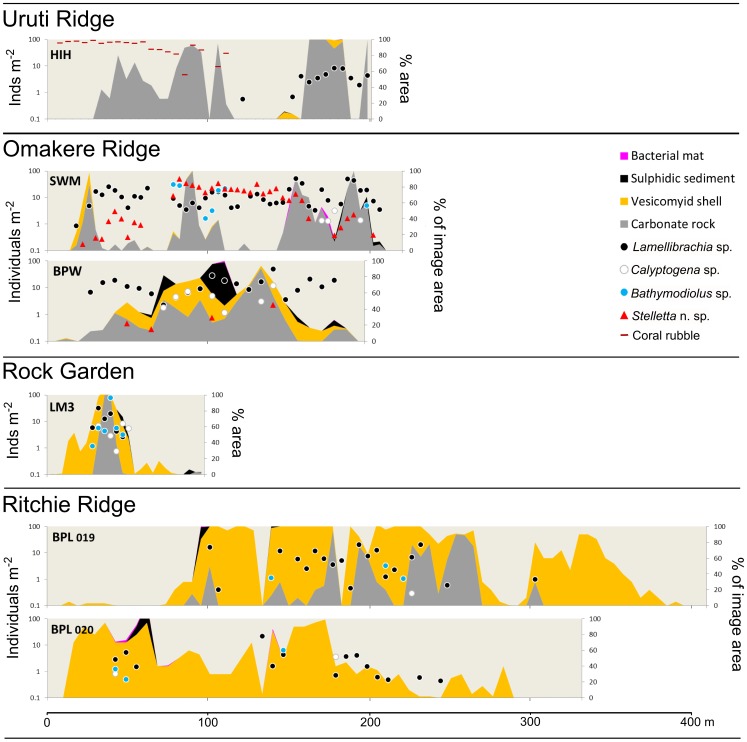
Representative profiles across selected seep sites at Uruti Ridge, Omakere Ridge, Rock Garden, and Ritchie Ridge. Site name abbreviations: HIH, Hihi; SWM, Southwest Moa; BPW, Bear's Paw; LM3, LM-3; BPL, Builder's Pencil (figures on Builder's Pencil profiles show station numbers. Station numbers for other sites: HIH, TAN0616-066; SO214-094; BPW, SO191-52-3; LM3, SO191-042). Data, symbols, and scale as for Figure 5.

*Hihi*, *LM-10*, and *Kereru*. No additional sampling has been undertaken at these sites since TAN0616. Habitats and fauna have been described by Baco et al. [8]. All three sites consist primarily of extensive weathered carbonate rock platforms with sparse *Lamellibrachia* sp. populations close to the flare co-ordinates, scattered *Calyptogena* sp. shell fragments, and some patches of bacteria-covered rock (Figure 6). Scleractinian coral rubble and evidence of trawling (abandoned gear and scour marks) were widespread.

#### Omakere Ridge

*LM-9*, *Kea and Kaka*, *Bear's Paw*, and *Moa*. Other than a single multicorer deployment at Bear's Paw during SO214 (SO214 station 095), no additional biological sampling has been undertaken at these sites since SO191. Substratum and faunal characteristics have been described by Jones et al. [14] but re-analysis of images here showed that *Stelletta* n. sp. sponges were present on carbonates at all sites, and that bathymodiolin mussels were present in low densities at all sites except LM-9.

*Moa (reef)*. As described by Jones et al. [14], this is an area of massive, weathered, carbonate rock colonised by sessile heterotrophic fauna including scleractinian and antipatherian corals, and sponges. No live seep-associated fauna were recorded.

*Southwest Moa*. Jones et al. [14] noted that the south-western extension of Moa had very different physical and faunal characteristics to the main part of Moa. Additional SSS coverage collected during SO214 together with a single camera transect across the western extremity of the site confirmed that Southwest Moa is an area of active seepage supporting the highest densities of chemoautotrophic taxa recorded at any site. (Figure 2 E, Figure 6). *Stelletta* n. sp. sponges were particularly abundant and, together with *Lamellibrachia* sp. tubeworms commonly occurred on the upper surfaces of carbonate blocks and pavements, in contrast to most other sites where fauna rarely projected above the upper surfaces of the carbonates.

#### Rock Garden

*Rock Garden Knoll*. Extensive weathered carbonate substratum was recorded but with no seep fauna recorded other than a single live *Lamellibrachia* sp. tubeworm.

*Faure Site*. As described by Naudts et al. [34], transects showed primarily muddy sediments with *Calyptogena* sp. clam shells in some areas, low densities of *Lamellibrachia* sp. tubes, and some patches of dark sediments. Still images were intermittent and of low quality at this site but video showed apparently live Bathymodiolin mussels associated with one of the areas of clam shell.

*Weka*. Image quality here was also poor but video revealed extensive areas of weathered carbonate platform with widespread *Calyptogena* sp. shells and, at the Weka *a* site, occasional *Lamellibrachia* sp. tubes. No live seep fauna were recorded.

*LM-3*. As described by Naudts et al [34], the only camera transect across this site showed extensive weathered carbonates with a single small patch (ca. 25 m across, Figure 6) with very high densities of chemoautotrophic fauna including Bathymodiolin mussels, *Lamellibrachia* sp., and *Pseudosuberites* sp. (Figure 6).

#### Ritchie Ridge

*Builder's Pencil* is on a ridge of weathered carbonates and is remarkable for extensive areas of seabed overlain by accumulations of *Calyptogena* sp. clam shells. Video analyses confirmed a seabed area of ca. 70,000 m^2^ covered by clam shells (*cf* [8]), many of which were worn and had brown Fe–Mn oxide discolouration characteristic of long-term presence on the seabed. Live *Lamellibrachia* sp. tubeworms were widespread but sparse, reaching maximum recorded densities of 21 individuals m^−2^ and were always either in close contact with the substratum or in crevices and under ledges. Bathymodiolin mussels occurred frequently but at low densities, and only one patch of live *Calyptogena* sp. clams was recorded. On the flanks of the shell-covered ridge, carbonate rocks were colonised by sponges, crinoids, and antipatherian, stylasterid, and gorgonian corals.

### Comparisons among sites

Profiles across seep sites in each of the regions, using data from analysis of still images illustrate differences in substratum and community composition within and among regions (Figures 5 and 6). Conspicuous distinctions among sites are the absence of carbonate rocks at Takahe, the extensive areas covered by *Calyptogena* sp. shells at Builder's Pencil, and the high faunal densities at Omakere Ridge sites, including *Stelletta* n. sp. which was recorded only in this region.

**Figure 7:**
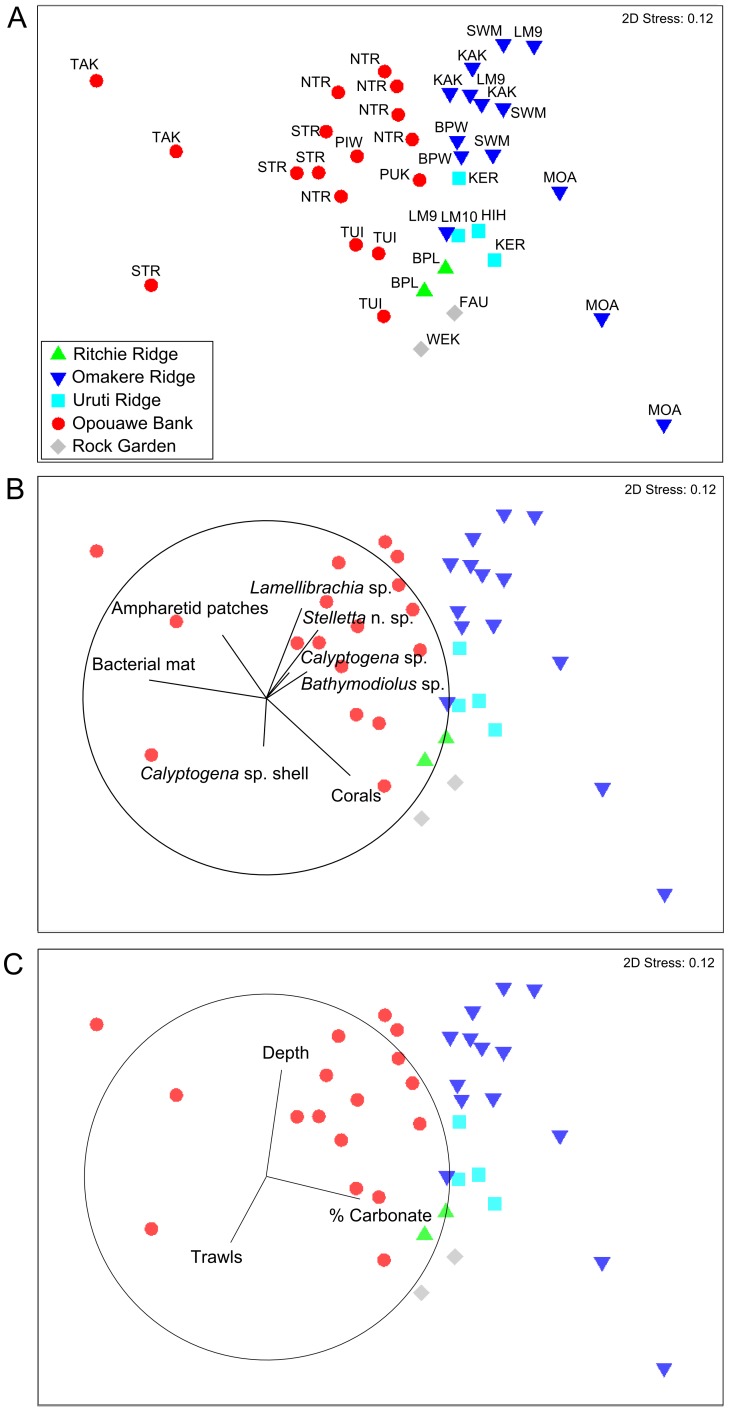
Non-metric multivariate scaling (NMDS) ordination of Hellinger distances between seep communities observed in video transects. Underlying data are log-transformed occurrence records of chemoautotrophic megafauna (*Lamellibrachia* sp. tubeworms, *Calyptogena* sp. clams, *Bathymodiolus* sp. mussels, *Stelletta* n. sp. sponges), seep-associated substrata (bacterial mats, ampharetid patches and *Calyptogena* sp. clam shells), and intact scleractinian coral matrix. A – Transects distinguished by region (symbols; see Figure 1 for context) and seep site (labels; see Table 1 for full names). B – Contributions (Pearson correlations) of faunal variables to between-sample distances. C – Relationships (Pearson correlations) between three environmental variables and the distribution of transects in the ordination: depth (−0.57); % carbonate rock substratum (0.52), and number of trawls per site from 1998 to 2005 (0.41).

The NMDS ordination based on observations of seep-associated fauna and corals in video transects (Figure 7) indicated that communities range between three extreme states represented by transects at Takahe, Southwest Moa, and Moa Reef, respectively. The Takahe extreme was characterised primarily by the absence of all mega-epifauna other than highly localised populations of *Calyptogena* sp. clams associated with small areas (m^2^ scale) of disarticulated shell, and the presence of bacterial mats and ampharetid patches. The Southwest Moa extreme was characterised by the highest densities of all seep-associated taxa, and moderate areas of *Calyptogena* sp. shell. The third extreme, represented by Moa, was characterised by scleractinian corals and low densities, or absence of, chemoautotrophic taxa, including ampharetid patches and bacteria. Vectors of variable contributions (Figure 7 B) show that variability among sites along an axis from top left (Takahe) to bottom right (Moa) in the ordination was driven primarily by changes in the relative occurrence of bacterial mats and ampharetid patches (increasing towards top left), and scleractinian corals (increasing towards bottom right). However, there was also marked variability along an axis orthogonal to this which was associated primarily with changes in the occurrences of live populations of the four megafaunal chemoautotrophic taxa; occurrence of clams, tube worms, mussels, and *Stelletta* n. sp. sponges decreasing from upper right to bottom left. The horizontal dimension of the ordination was correlated with the percentage of carbonate rock substratum (Pearson correlation, 0.52); carbonates increasing from the Takahe to the Moa extremes (Figure 7 C). The vertical dimension was correlated with both depth (−0.57) and trawling (0.41); deepest sites being at the top of the ordination, and number of trawls per site increasing from the South-west Moa extreme towards the lower left of the ordination.

The spread of data points between the three extreme states in the ordination indicated considerable variability at both within-region and within-site scales. For instance, Opouawe Bank sites span a large proportion of the total ordination space, ranging from the extreme, no-carbonate, no-tubeworm, no-mussel state of Takahe to massive carbonates with large areas of *Calyptogena* sp. shells at Tui, and moderate to high densities of tubeworms and carbonates at North Tower. Similarly, in the Omakere Ridge region, Moa and Southwest Moa are adjoining sites yet contain strongly contrasting habitats and fauna; transects at these sites representing two of the three extreme states in the ordination.

The ordination also indicated some separation between regions, particularly between Opouawe Bank and the other regions. This was driven in part by higher incidence of bacterial mats and ampharetid patches at Opouawe Bank sites, but also by the presence of *Stelletta* n. sp. sponges at most Omakere Ridge sites, and corals at Moa and the Uruti Ridge sites. However, there was convergence between sites independent of spatial separation. For instance, Tui and Builder's Pencil were similar, despite being located on Opouawe Bank and Ritchie Ridge, respectively; the similarity in this case being driven largely by high proportions of seabed covered by *Calyptogena* shells at both sites.

### Assessing impacts of human activities

#### Trawling

**Figure 8:**
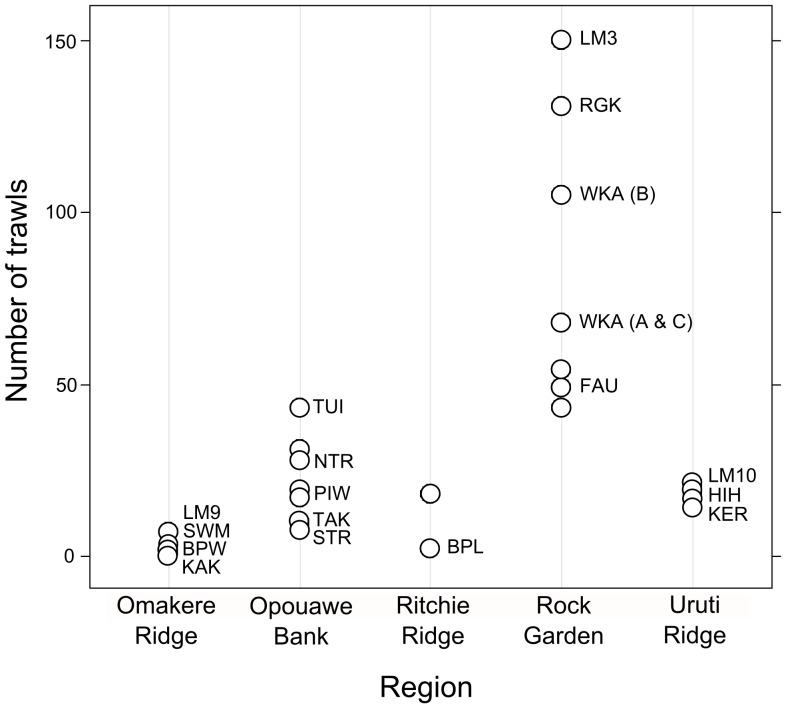
Trawl intensity at seep sites by region. Selected seep sites are identified by name (see Table 1 for full names). Trawl data are cumulative totals for the period 1989–90 to 2004–5 [24] calculated as the number of trawl tracks intersecting a 250 m radius circle around seep site positions given in Greinert et al. [12].

Sites in the Rock Garden region (Rock Garden Knoll, Faure Sites, Weka, and LM-3) were the most heavily trawled, with cumulative trawl numbers for the period 1998–2005 ranging from 43 to 54 trawls at Faure Site up to 150 trawls at LM-3 (Figure 8). In the Opouawe Bank region, the number of trawls ranged from eight at South Tower to 43 at Tui, with 28 at North Tower. Sites on Uruti Ridge (Hihi, LM-10, and Kereru) were each trawled ca. 20 times. Omakere Ridge sites were least impacted by trawling, with seven trawls at LM-9, one at Kea and Kaka, one at Southwest Moa, and three at Moa. Comparing these values to the distribution of sites in the NMDS ordination (Figure 7 C) showed that increasing trawl intensity was associated with the gradient of decreasing occurrence of live chemoautotrophic fauna identified above, but was also inversely correlated with depth; shallower sites having fewer live fauna and greater trawl intensity.

#### Gas hydrate extraction

**Figure 9:**
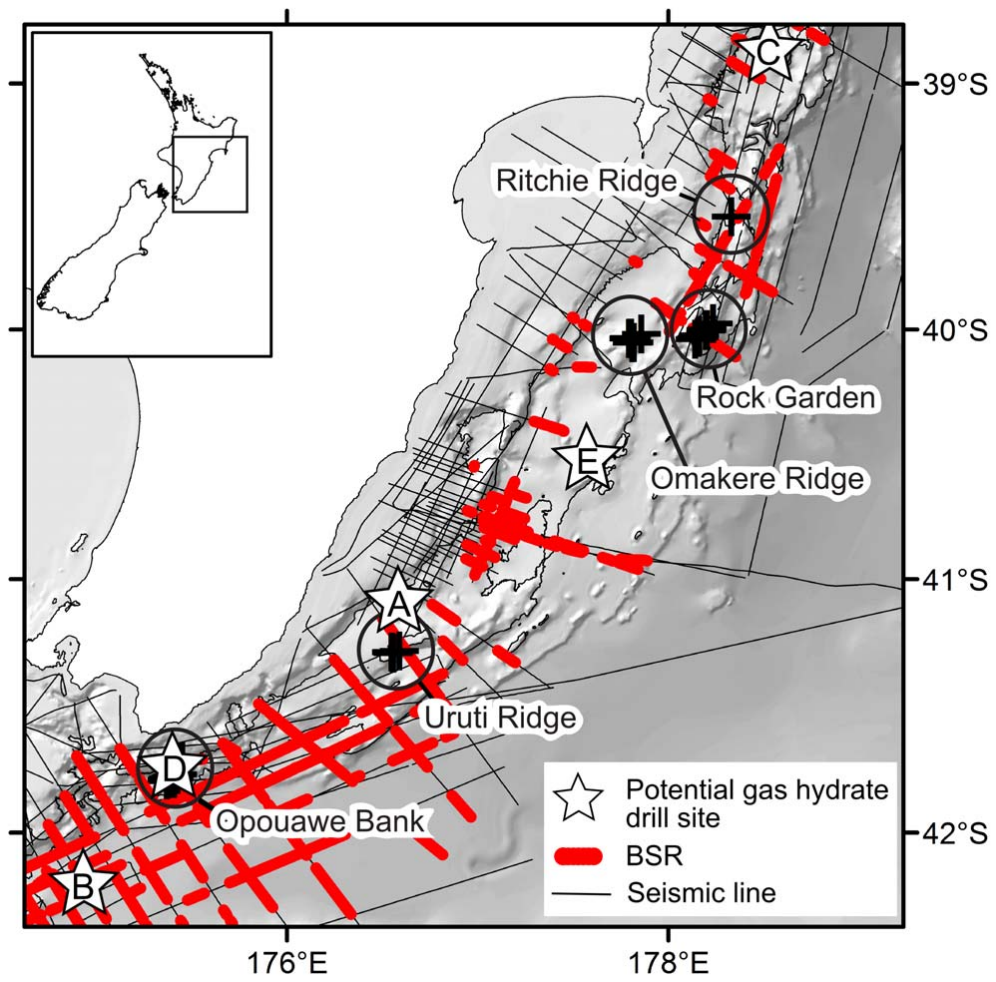
Cold seep communities and gas hydrates. Distribution of cold seep faunal communities (black crosses, regions as in Figure 1) on the Hikurangi Margin in relation to occurrence of bottom simulating reflectors (BSR) in seismic survey data and sites of potential interest for gas hydrate exploration (white stars): A, channel system north of Uruti Ridge; B, Pegasus Basin; C, Tuaheni Basin; D, Opouawe Bank; E, Porangahau Ridge.

Seismic surveys of the Hikurangi Margin [20], [25]–[28] have shown that bottom simulating reflectors (BSRs), which are indicative of free gas underlying gas hydrates, are widespread across much of the margin (Figure 9). Recent interpretations suggest that most BSRs on the margin represent gas hydrates in fine-grained sediments and mudstones [25] which are unlikely to be suitable for commercial hydrate extraction [35]. The seep sites are associated with stronger BSRs in areas where methane-rich fluids pass through the gas hydrate stability zone (GHSZ; where pressure and temperature conditions favour hydrate formation) and reach the seabed either via direct vertical ‘chimneys’ or by migration along stratigraphic pathways and emergence through networks of extensional faults [26], [36]. Current resource interest centres on hydrates and free gas reserves in sand reservoir strata, which are not necessarily associated with BSRs [35]. These strata have not yet been well-defined across the Hikurangi Margin [37] and thus the likely locations of any future extraction of hydrates in relation to the seep communities are not yet known. A number of sites have been proposed for research drilling, however, including Opouawe Bank and sand channel systems north of Uruti Ridge [38], both of which would be in close proximity to known chemoautotrophic communities (Figure 9).

## Discussion

### Habitat and community composition and structure

Study of the Hikurangi Margin cold seep sites, primarily using towed camera systems but augmented with information from corer, grab and epibenthic sled sampling [8], [12], [14]–[18], has enabled description of the principal chemoautotrophic mega-fauna present, their population densities, the physical habitats they are associated with, and how these parameters vary across the study area. The small spatial scale of seep microhabitats in relation to the intensity of sampling to date makes it likely that isolated patches of live fauna will have been missed at any given site, but broad conclusions concerning distributions of fauna and substrata are well-supported. For instance, intensive sampling with a range of methods at sites on Opouawe Bank did not record the distinctive spherical sponge *Stelletta* n. sp., which was abundant at sites in the Omakere Ridge region, nor the scleractinian corals that were recorded at Moa and at the Uruti Ridge sites. Similarly, the relative extents of different seep-associated substrata described here, including authigenic carbonates, sulphide-rich sediments, and clam shells, are likely to be reliable, particularly across the Opouawe Bank and Omakere Ridge regions where full SSS coverage provides accurate measures of seep site extent.

The seep communities on the Hikurangi Margin share some characteristics with those from both seeps and vents in other parts of the world [3], [7], [39]–[45], including dominance of mega-epifaunal communities by large siboglinid tubeworms, vesicomyid clams, and bathymodiolin mussels, but there are also differences in the combinations of fauna in different regions. For instance, while the well-oxygenated New Zealand seep sites [16] are characteristically populated by mixed communities of tubeworms, clams, mussels, and sponges, those on the Californian and Oregon margins are dominated by bacterial mats and vesicomyid clams within the oxygen minimum zone [44] but by tubeworms and clams in oxygenated areas [45]. In the Atlantic, seeps in the Gulf of Mexico and on the equatorial West African margin are dominated by tubeworms and mussels, although clams may also be present [39], [42]. Perhaps more significantly, the distinctive ampharetid beds appear to be ubiquitous at the New Zealand sites. Although these beds are not unique to the New Zealand region, similar tube-building ampharetids having been observed around vesicomyid clam beds at Hydrate Ridge [18], their density, biomass, and frequency of occurrence are apparently greater on the Hikurangi Margin ([18], AT and LL unpublished data). Thus there appear to be structural differences between the seep communities of the Hikurangi Margin and those elsewhere in the world.

#### Inter-site differences

Differences among sites were associated with variations in the relative abundances and presence or absence of taxa, and the types and proportions of seep-associated substrata present. While there was some distinction by region, most obviously with the presence of *Stelletta* n. sp. only at Omakere Ridge sites, most taxa and most habitats were observed in most regions. There was, however, wide variability within regions in terms of seep size, dominant substrata, and community composition. We suggest that this variability is driven (1) by the ages of the seeps (i.e. the history of fluid flow at each site) and thus the successional stage of the faunal communities inhabiting them, and (2) by the history of disturbance from trawling. We expand on these points below, starting with evaluations of the ecology of the principal chemoautotrophic fauna and their likely roles in succession.

#### Succession

Although Levin [4] highlighted the need for *in situ* measurements over extended periods for developing full understanding of successional processes at cold seeps, a growing literature on the trophic and reproductive ecology of the principal seep megafaunal taxa provides a logical framework around which to develop a successional model for the Hikurangi Margin seeps (as has been done for whale falls [46] and later stages of Gulf of Mexico seeps [33]). This is an important goal because understanding of the sequence and rate of succession is essential for evaluating the vulnerability of these sites to present and potential future disturbances. We do not yet have life-history information for any of the New Zealand megafaunal taxa but detailed studies of congeneric species of *Lamellibrachia* [47]–[51] and *Calyptogena* [52]–[56] in other seep provinces, when combined with inferences from *in situ* observations here, enable us to construct a first hypothetical model of succession for the Hikurangi seeps.

From their ubiquitous presence in Hikurangi Margin seep environments and what is known of their chemistry and ecology [16]–[18], [31], [57], it is likely that the ampharetid populations play a part in the early stages of seep community development. Sommer et al. [16], [31] have speculated that the ampharetids play an important role in early successional stages by increasing the concentration of sulphides in surficial sediments. Their hypothesis suggests that tube building by the ampharetids actively promotes growth of the bacteria they feed on by providing conduits that simultaneously increase rates of methane flux through the sediment-water interface and concentrations of sulphate-rich seawater in near-surface sediments. Together, these processes would also promote increased rates of microbial anaerobic oxidation of methane (AOM, [58]) beneath the ampharetid patch and thus explain the higher sulphide concentrations measured in ampharetid patches than in surrounding sediments [16]. Regardless of the actual pathways involved, measurements of much higher rates of oxygen consumption, methane flux, and sulphide concentrations within ampharetid patches [16] support a hypothesis that the ampharetid populations modify habitats in a way that increases the availability of sulphide and methane at the seabed, thus facilitating seep community development.

Sommer et al. [16] and Dale et al. [57] have argued that ampharetid beds are a transitional stage in seep development between the onset of fluid flow and the establishment of bacterial mats. Examination of seabed images and consideration of the trophic ecology of the ampharetids, however, suggests that the sequence might be the reverse of this; bacterial mats preceding establishment of ampharetid populations. The morphology and stable isotope signatures of the ampharetids show that they are heterotrophic and that most of their carbon intake is derived from aerobic methanotrophy; probably by direct consumption of methane-oxidising bacteria [17], [18]. This suggests that the ampharetids recruit to patches in which their food, bacteria, is abundant. Furthermore, seabed images show that bacterial mats often occurred on sediments where there was no sign of associated ampharetid pits or colonisation by any other seep-associated metazoans.

The *Calyptogena* sp. clams have thiotrophic symbionts [17] and in this study live clams were only observed embedded in dark, sulphide-rich, sediments colonised by ampharetid polychaetes. We suggest, therefore, that the clams recruit preferentially to patches that have first been ‘conditioned’ by the activities of ampharetid polychaetes to have high sulphide concentrations. While this might not be an obligatory relationship, our observations suggest a hypothesis that the presence of ampharetid populations facilitates clam settlement by enhancing sulphide availability. From this study we know that adult *Calyptogena* spp. clams are ∼40 to 80 mm long, live partially buried in the sulphide-rich sediment patches and, based on the ratio of disarticulated shells to live populations, apparently experience high rates of mortality. Congeneric vesicomyids also have thiotrophic symbionts [1], [59], can have relatively rapid growth (*C. kilmeri*; 80% of asymptotic length at 6.6 y, [52]), and can exhibit near-continuous reproduction [54]; characteristics that are associated with taxa in ephermeral habitats [60], [61]. Although both slower growth [56] and indirect evidence for seasonal reproduction [55] have been reported, these observations set bounds on habitats that *Calyptogena* spp. can colonise and suggest a minimum necessary habitat persistence time: they require soft sediments with high concentrations of sulphide accessible within ∼80 mm of the sediment surface, which persist for at least 5 to 10 years (i.e. the shortest generation time reported for a congeneric [52]). Thus, in combination with our observations that live clam populations are small and highly-localised, and that areas of disarticulated shells are widespread, we might characterise *Calyptogena* spp. clams on the Hikurangi Margin as early colonisers of potentially short-lived (years to decades) sulphide-rich surficial sediment patches. Over short time scales (years) cessation of fluid flux in a given patch will result in extinction of the local (m^2^ scale) population as sulphides are depleted. Over much longer time scales (100 s–1000 s y) capping of fluid flow by accumulation of carbonate precipitates will have the same effect over larger spatial scales (ha scale). This expectation matches the common observation of disarticulated shells, often in very large numbers, in the present study but leaves us with a poorly-constrained idea of the actual timescales involved. Thus, while it is possible that the quantity of accumulated shell material at a site is correlated with the local history of fluid flow, and thus seep age, we cannot yet put bounds on this on the basis of the clams alone.

By introducing seawater, and thus sulphate, into sediments, the clams also enhance AOM [62]–[64]. Over long time scales, if fluid flux to the patch persists, locally-enhanced rates of AOM result in the build-up of authigenic carbonate particles in surficial sediments [65], thus generating a local environment which is rich in sulphide and methane but which also has hard substrata. These are presumably the conditions required for recruitment by *Lamellibrachia* tubeworms, bathymodiolin mussels, and the seep-associated sponges, all of which need hard substrata for settlement.

The lamellibrachid tubeworms have thiotrophic endosymbionts (but see [17]) and their tubes attach to, and extend deep within, carbonate rock concretions. *Lamellibrachia luymesi* tubeworms at cold seeps in the Gulf of Mexico are of comparable size to the New Zealand species [51] and are extremely long-lived (100 s y) [46]–[49]. There is also persuasive evidence that *L. luymesi* actively promotes AOM, and thus the continued generation of sulphides, around its ‘root’ by releasing sulphate into sediments beneath carbonate concretions [66]–[68]. If the Hikurangi Margin species has similar characteristics, the primary constraints on colonisation for the tubeworms will be the availability of sulphides for energy, hard substrata for settlement, and established methane-rich fluid flow over extended periods (10 s–100 s y). Thus, while the tubeworms exploit the same energy resource (sulphide) as the clams, their attachment to hard substrata, longevity, and ability to access, and promote, generation of sulphides deeper in the sediments indicate recruitment at a later successional stage and very different life-history characteristics. They colonise only when fluid flow has been established long enough for carbonate precipitates to form, providing substrata for settlement, but may persist for as long as methane flux to sediments beneath the accumulating carbonate continues. That they might actively promote AOM in the sediments further suggests an important role in modifying physical habitats within the seep. Once established, increases in the rate and duration of AOM caused by the worms themselves will result in increased generation of carbonates, thus accelerating the development of carbonate chemoherm habitat.

The bathymodiolin mussels at the Hikurangi seeps rely primarily on methanotrophic symbionts [17] and thus exploit a different energy resource to the clams and tubeworms. Because they attach to hard substrata, however, they are dependent on availability of carbonates and thus will be later successional species than *Calyptogena* sp., recruiting to mature seep sites with carbonate substrata and methane flux to the water-column. Furthermore, because methane flux at seeps is variable across a range of spatial and temporal scales [15], [29], [34], rapid colonisation and growth, coupled with short generation times, are also likely characteristics [61].

Little is known about the ecology of the two sponge species common at the seep sites; *Pseudosuberites* sp. and *Stelletta* n. sp. but the former, at least, can support a diverse commensal macrofaunal community characterised by high levels of methane-derived carbon [17]. Thurber et al. [17] hypothesise that the sponge may be chemoautotrophic and play a significant role in facilitating transfer of methane into the metazoan food web in hard substrata habitats at the seep sites.

Finally, we also know that all chemoautotrophic taxa are ultimately dependent on the flux of methane-rich fluids into seabed sediments. Consequently, when flow to a site ceases completely, all chemoautotrophic populations will die out and conditions will become suitable for colonisation by heterotrophic fauna. On carbonates, these are likely to include sponges, corals, and other sessile heterotrophic epifauna. The transition to heterotrophic communities is a gradual process, however, as many ‘background’ slope taxa can be tolerant of sulphide and may colonise seep sites before fluid flow ceases [69].

Liebetrau et al. [29] used uranium–thorium (U–Th) dating to estimate the age of carbonate rocks at North Tower, LM-10, Bear's Paw, and Moa. Bear's Paw was the youngest site, with a maximum recorded age of 2,360±70 years before present, North Tower was older (4,950±650 ybp), while LM-10 was considerably older (12,400±160 ybp). At Moa, only surficial carbonates were sampled, yielding dates for the most recent carbonates of 4,390±130 ybp. The times of both the onset and the most recent seepage are likely to be relevant to the structure of the seep communities and habitats observed in the present. The time since onset will influence the extent and thickness of carbonates, and thus the types of habitat available for colonisation, while the time of most recent activity will influence whether present habitats are more suitable for chemoautotrophic or heterotrophic fauna. The youngest carbonates measured at each of these sites ranged from 2,090 to 4,390 ybp, with the sites ranked from oldest to youngest as Moa (4,390±130 ybp), LM-10 (4,120±40 ybp), North Tower (3,960±50 ybp), Bear's Paw (2,090±850 ybp). Obvious matches between this chronology and our observations are that ‘weathered’ carbonates colonised by cold water corals were present at Moa and LM-10, whereas ‘fresh’ carbonates colonised by high densities of chemoautotrophic fauna were present at North Tower and Bear's Paw. Furthermore, while the youngest carbonates at LM-10 are of similar age to those at North Tower, the oldest carbonates at LM-10 are much older, indicating a considerably longer history of fluid flow.

While the U–Th data provide useful indicative ages, it is also important to note that these dates relate to the consolidated upper layers of the carbonates. Beneath the carbonates, at the interface between carbonate blocks and the underlying sediments, the process of carbonate build-up via AOM continues in the present. Evidence for this comes from observations of calcite particles in anoxic sediments beneath a large block retrieved from North Tower during SO191 (A. Eisenhauer, IFM-Geomar and DB, personal observation – Figure 3 B), as well as the presence of live chemoautotrophic fauna, which are reliable indications of continuing seepage in the present.

**Figure 10:**
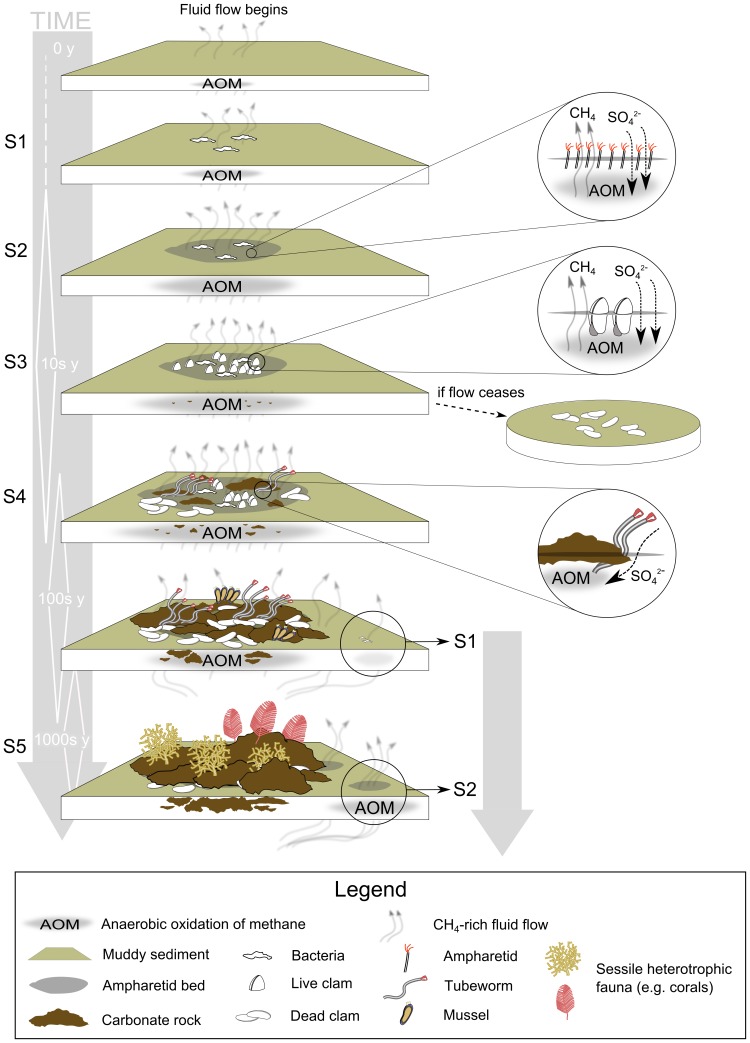
Hypothetical succession sequence at Hikurangi Margin cold seep sites. Labels ‘S1’, ‘S2’, etc., indicate Successional Stages as described in the discussion text.

Much of this reasoning about seep succession is necessarily speculative but many of the basic ecological constraints (e.g. soft vs. hard substrata, thiotrophy vs. methanotrophy vs. heterotrophy), as well as likely time scales (e.g. years to decades for establishment of ampharetid beds and *Calyptogena* populations vs. centuries for establishment of *Lamellibrachia* populations vs. millennia for development of carbonate reefs) are likely to be reliable. Putting these bounds and processes together, we propose a hypothetical sequence of succession at Hikurangi Margin cold seep sites consisting of ten principal steps which result in five successional stages identifiable from seabed observations (Figure 10). Dates are poorly constrained in this scheme but indicative ranges are given as years since the onset of fluid flux in a patch, based on literature values for geochemical and ecological processes as discussed above:

1. Onset of **localised flux of methane-rich fluid** from deeper strata to the sediment-water interface [20], [36].
2. **Anaerobic oxidation of methane** by microbial consortia within sediments generates patches of sulphide close to the sediment surface [58], but most methane continues to reach the sediment-seawater boundary layer [57].
3. Availability of methane and sulphide within the patch promote growth of **aerobic methanotrophic and thiotrophic microbial communities** at the sediment surface [*Succession Stage 1: colonisation by aerobic microbial community – ca. 1–10 y*]
4. Heterotrophic **ampharetid polychaetes** colonise the patch, feeding on abundant microbial production, primarily by aerobic methanotrophic microbes at the sediment surface [17], [18], [57].
5. Tube building by **high-density ampharetid populations** increases permeability of sediments and thus both upward flux of methane-rich fluids through the patch and downward irrigation of sulphate-rich seawater into the sediments. This increases both aerobic microbial production at the sediment surface and AOM within the sediments [16], [57]. [*Succession Stage 2: Colonisation by ampharetid polychaetes – ca. 1–100 y*]
6. Locally elevated supply of sulphide within the patch facilitates colonisation by ***Calyptogena*** **clams** (and other vesicomyid taxa), which require high sulphide concentrations in surficial soft sediments. Clams also contribute to irrigation of sulphate-rich seawater into sediments, further enhancing AOM [62]–[64] *[Succession Stage 3: Colonisation by clams – ca. >50 y]*.
  - If flow ceases in patch, local die-off of ampharetids and clams follows and disarticulated clam shells accumulate on background sediment.
7. If flow persists, **authigenic carbonate precipitates** form hard substratum particles in the patch, enabling colonisation by ***Lamellibrachia*** **tubeworms** (thiotrophic symbionts). [*Succession Stage 4: Carbonate precipitates enable colonisation by lamellibrachid tubeworms – ca. >100 s y*]
8. Continuing build-up of carbonate modifies flow and reduces the extent of sulphide-rich sediments. This causes local reduction of *Calyptogena* populations but favours recruitment of **Bathymodiolin mussels** (requiring hard substrata and methane flux to the water column)
9. Long lived *Lamellibrachia* (100 s y [51]) continue to access deep sulphide below carbonates through their ‘root’, stimulating continued sulphide and carbonate generation though AOM by actively introducing sulphate to deep sediments [66]–[68].
10. **Carbonate build-up continues** and eventually caps the original seep site, causing flow to be displaced to periphery of carbonate platform. Widespread failure of clam populations and more gradual decline of tubeworm populations follows before carbonate rocks are colonised by **non-seep epifauna**; corals, non-methanotrophic sponges, etc. [*Succession Stage 5: Colonisation by non-chemosynthetic epifauna – ca. >1000 s y* [29]].

### Vulnerability to human activities

**Figure 11:**
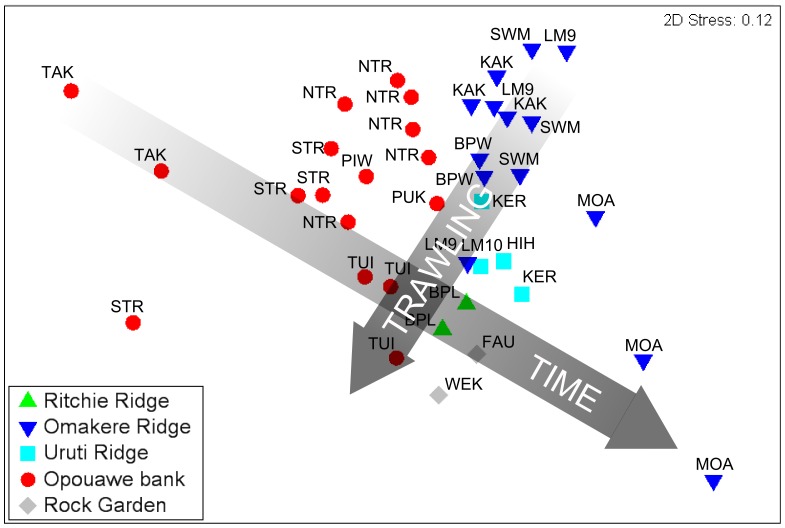
Interaction between seep site age and disturbance from trawling. NMDS ordination of seep site communities (see Figure 7) showing conceptual illustration of the relative influences of seep site age and trawling on observed seep characteristics.

Anthropogenic threats to the seep sites include on-going physical disturbance from bottom trawling, and potential modification of fluid flow patterns resulting from future large-scale extraction of methane hydrates. While hydrate extraction is still in the resource evaluation phase, the trawl frequency data here show that most sites were affected by bottom trawling in the period from 1989 to 2005, with trawl intensity ranging from a single trawl at some Omakere Ridge sites to 150 trawls at LM-3 (Figure 8). Despite the wide variability in trawl intensity between regions and sites, detecting quantifiable effects of trawling on the seep fauna is not straightforward for two reasons. First, any effects of trawling are likely to be overlaid on differences in successional stage (i.e. the age of the seep site) outlined above (Figure 11). Thus, while South Tower, Takahe, and Hihi have similar trawl histories, any visible effects of trawling are likely to differ between the low-lying authigenic carbonate habitats at South Tower by comparison with the open muddy sediments at Takahe, or the massive late-stage carbonate platforms at Hihi. Second, the heterogeneous physical structures within carbonate concretions are likely to create localised refugia in which benthic fauna escape trawl impacts. An example of this is the thriving but highly localised (ca. 25 m diameter) chemoautotrophic community observed at LM-3, despite this site having the highest recorded frequency of trawling.

There are, however, qualitative differences between regions that might be related to differences in trawling history. Most obviously, very few live chemoautotrophic taxa were recorded at sites in the Rock Garden region by comparison with any of the other regions: apart from sparse *Lamellibrachia* sp. tubeworms at Rock Garden Knoll and Faure Site, and the single patch of seep fauna at LM-3, only disarticulated clam shells were recorded. It is also clear that *Stelletta* n. sp. sponges were seen, often in high densities, only at sites in the Omakere Ridge region, where trawl intensity was lowest. Also apparent from close examination of still images in particular, are differences in the growth forms of lamellibrachid tubeworms. At most sites, *Lamellibrachia* tubes emerged from beneath carbonate blocks and ledges and characteristically grew along overhangs or in the spaces between blocks [8]. At the least-trawled sites, Southwest Moa, Bear's Paw, and Kea-Kaka, however, tubeworms grew directly from the upper surfaces of carbonate blocks (Figure 2 D), often in association with *Stelletta* n. sp. sponges. Finally, although not a seep-associated taxon, matrix-forming scleractinian corals were seen as intact ‘thickets’ only at Moa in the Omakere Ridge region (Figure 2 F), whereas on similar rock substrata in the heavily trawled Rock Garden and Uruti Ridge regions, extensive areas of broken fragments of coral were recorded (Figure 6).

Based on their physical form and what is known of their life histories, we can construct a ranking of the principal chemoautotrophic taxa in terms of their likely vulnerability to physical disturbance. *Calyptogena* sp. clams exploit transient sulphide patches, suggesting that their populations may be resilient to intermittent disturbance. However, if, as proposed above, the establishment of dense ampharetid populations is important for generating high-sulphide patches, it is conceivable that the viability of clam populations might be influenced by the resilience of ampharetids to disturbance, as well as that of the clams themselves. Clams potentially reach reproductive maturity in less than five years from first settlement [52] but Dale et al. [57] estimate that the ampharetid patches they studied at North Tower to be in the order of 70 years old. Thus, if the ampharetids were to be particularly susceptible to disturbance, whether from direct contact or from smothering by resuspended sediments, this could have consequences for clam populations over longer time scales through reduction in the availability of sulphide-rich habitat.

Lamellibrachid tubeworms apparently gain some protection from physical impacts by virtue of their habitat within the authigenic carbonates of the seep. However, if the high population densities and distinctive growth form of tubeworms at Southwest Moa and other Omakere Ridge sites are representative of undisturbed populations, it seems likely that populations at all other sites have been affected by trawling. The chitinous material of their tubes is very tough, requiring considerable force to cut or break in recovered specimens (authors' personal observations and [32]), yet splintered and broken tubes were recorded in several transects. While physical impact is the most likely cause of such breakage, no well-defined correlation with trawl intensity was apparent in the present data, other than the observation that tubeworm populations were sparse, highly localised, or absent at sites where trawling was most intense. Observations of live tubeworms in moderate densities at Uruti Ridge sites where there was obvious evidence of trawl damage in the form of coral fragments, trawl marks, and abandoned trawl gear [8], suggest that they are more resilient to this kind of impact than sessile suspension-feeding taxa such as corals and sponges. While persistence of chemoautotrophic communities at even heavily trawled sites might suggest that some of the seep fauna are resistant to physical disturbance, existing data are not sufficient to determine the extent to which whole communities might have been removed, nor yet the longer-term consequences of on-going disturbances. It is clear, however, that coral-dominated communities characteristic of later successional stages at the seep sites are highly vulnerable to direct physical impacts and have long recovery times.

Techniques for large-scale extraction of seabed gas hydrates have not yet been developed and target sites have yet to be identified with any confidence. However, potential impacts on seep communities can be classified into four categories: (1) direct physical disturbances of the seabed; (2) smothering by re-suspended sediments or tailings, (3) indirect effects associated with modification of fluid flow to the seep sites, and (4) large-scale destabilisation of slope sediments. It is currently considered unlikely that extraction would directly target the anticlinal structures on which many seep communities are found, because of their geological complexity and consequent unpredictability of flow [35], and considering the potentially catastrophic consequences of slope destabilisation, its prevention is likely to be a major factor in extraction planning. If this proves to be the case, direct physical impacts and smothering might represent lower levels of risk to seep communities than the consequences of flow modification.

Any reduction in fluid flow to sites of established seep communities would affect all successional stages proposed here, with consequent declines in the populations of all chemoautotrophic taxa. For any future large-scale resource extraction, therefore, the potential ecological consequences would need to be assessed by consideration of the specific geologic setting of the hydrate reserves, the proximity of extraction sites to seep sites with live chemoautotrophic communities, the spatial scale over which flow regimes would be affected, and thus how flow at neighbouring seep sites would be affected. A strategy to manage impacts would require consideration of the life-history characteristics of the principal taxa and the time scales associated with each of the successional stages proposed here, which range from years to millennia. In particular, it will be important to develop understanding of spatial and temporal scales of connectivity among populations, and evaluate susceptibility to the different categories of disturbance.
